# Biomedical identify the drugs between primary osteoporosis and sarcopenia

**DOI:** 10.1097/MD.0000000000042975

**Published:** 2025-07-11

**Authors:** Zhangxin Chen, Jianxing Lin, Zhengjin Wang

**Affiliations:** a Zhangzhou Health Vocational College, Zhangzhou, China; b Department of Spine Surgery, Renmin Hospital of Wuhan University, Wuhan, China.

**Keywords:** bioinformatics, osteoporosis, potential drugs, sarcopenia

## Abstract

With the aging population, the prevalence of sarcopenia and primary osteoporosis is increasing. However, the underlying mechanisms linking these 2 diseases remain unclear. The study aims to identify potential genes and pharmacological targets associated with both diseases through bioinformatics analysis. Datasets GSE35958 (primary osteoporosis) and GSE1428 (sarcopenia) were sourced from the gene expression omnibus database. Differentially expressed genes common to both conditions were identified, and functional roles were elucidated using gene ontology and Reactome pathway analyses. Protein–protein interaction networks and hub genes were analyzed using Cytoscape with the molecular complex detection plugin. Key gene expression profiles were validated by quantitative real-time PCR, and potential gene–drug interactions were explored using the drug–gene interaction database. The identified pharmacological agents contributed to the formulation of therapeutic strategies for both diseases. Our study identified 80 commonly expressed genes through the gene expression omnibus database. Gene ontology enrichment analysis was employed to categorize these genes into biological processes, cellular components, and molecular functions. Additionally, 6 Reactome pathways were identified (*P* < .05), leading to the characterization of 65 genes. The study conducted a protein–protein interaction analysis and employed molecular complex detection to evaluate a set of 65 genes, ultimately identifying 5 central genes. In this investigation, cancellous bone and muscle tissues from elderly individuals with osteoporosis and sarcopenia were subjected to quantitative real-time PCR validation. The analysis revealed that 5 genes were down-regulated in muscle tissue, whereas AXL, ERBB2, and GAB1 were up-regulated in bone tissue. Subsequently, these 5 genes were analyzed for drug–gene interactions, resulting in the identification of 13 Food and Drug Administration-approved drugs with potential therapeutic applications for osteoporosis and sarcopenia. The aforementioned 5 genes (AXL, GAB1, ERBB2, NRP2, and ESR1) along with 13 pharmacological agents represent promising candidates for enhancing the treatment of osteoporosis and sarcopenia. Investigations into gene–drug correlations and analyses of potential pharmacotherapies offer novel insights for drug repurposing and the exploration of therapeutic pathways.

## 1. Introduction

As society ages, the incidence of osteoporosis has become increasingly prevalent among the elderly population.^[1]^ This condition is characterized by a gradual loss of bone mass with advancing age, a process particularly pronounced in postmenopausal women due to the accelerated rate of bone loss associated with a marked decline in estrogen levels. Among individuals aged 60 years and older, the prevalence of osteoporosis is significantly higher in women compared to men.^[2]^ This condition predisposes the elderly to increased bone fragility, whereby even minor external forces, such as routine falls or coughing, may precipitate fractures. Elevated blood glucose levels and diabetes are associated with an increased risk of fragility fractures.^[3]^ These fractures not only result in physical discomfort among the elderly but also significantly impair their quality of life, while contributing to heightened rates of disability and mortality.^[4]^ A substantial number of older adults suffer from osteoporosis, yet timely detection and diagnosis often remain elusive. This challenge is partly attributable to the subtlety of early symptoms, which may manifest merely as mild back pain and are therefore frequently disregarded. Additionally, a lack of awareness regarding the importance of regular bone density assessments further contributes to the delayed identification of the condition. Sarcopenia, a prevalent health issue among the elderly population, is characterized by a progressive decline in muscle mass and strength, resulting in diminished physical functioning, including challenges with ambulation, impaired balance, and an increased risk of falls. Furthermore, sarcopenia is intricately linked with other geriatric syndromes, such as frailty and disability, which significantly compromise the independence and self-care capabilities of older adults. The absence of specific diagnostic criteria and the limited awareness of sarcopenia among the general population contribute to a significant number of older adults remaining unaware of their condition, thereby failing to pursue appropriate interventions.^[5]^

Sarcopenia and osteoporosis frequently co-occur in the elderly, yet they are often under-recognized, leading to potentially severe consequences.^[6,7]^ Pharmacological treatments for osteoporosis include bisphosphonates,^[8]^ such as alendronate^[9]^ and risedronate.^[10]^ These pharmacological agents enhance bone density by inhibiting osteoclast activity and decreasing bone resorption. It is essential for these medications to be administered on an empty stomach, and patients should remain in an upright position for a period following ingestion to mitigate the risk of gastrointestinal adverse effects.^[11]^ Calcitonin, including variants such as salmon calcitonin, not only inhibits osteoclast activity by binding to its receptor and activating intracellular signaling pathways, primarily involving cyclic AMP, but also possesses analgesic properties, making it particularly suitable for patients with osteoporosis who experience pain.^[12]^ Additionally, drugs that promote bone formation, such as Teriparatide (a parathyroid hormone analogue) stimulate osteoblast activity and facilitate the formation of new bone by activating the parathyroid hormone 1 receptor,^[13]^ which enhances Wnt/β-catenin signaling and increases the expression of osteogenic factors such as Runx2 and Osterix; however, the duration of use is typically limited to a maximum of 2 years due to potential risks of osteosarcoma with prolonged administration.^[14]^ Despite significant advancements, concerns persist regarding the rare side effects associated with these medications and the insufficient evidence supporting their long-term efficacy. Consequently, many patients who could potentially benefit from pharmacological intervention choose not to pursue it. Therefore, the clinical management of osteoporosis increasingly involves interventions targeting the gut–bone axis.^[15]^ Promising therapeutic strategies, such as fecal microbiota transplantation (FMT)^[16]^ and modulation of gut-derived metabolites,^[17]^ have been extensively investigated in recent studies. There remains a clinical imperative to devise strategies that enhance patient acceptance and adherence to these efficacious treatments, as well as to continue the development of new medications that minimize side effects and exhibit sustained anabolic effects on bone.

Although pharmacological interventions targeting sarcopenia are under investigation, contemporary research indicates that androgen analogues may play a role in male sarcopenic patients by modulating hormone levels to enhance muscle protein synthesis.^[18]^ Additionally, other pharmacological agents aim to ameliorate sarcopenia by modulating signaling pathways associated with muscle metabolism, such as the mTOR pathway; however, no specific drugs have yet achieved widespread clinical application.^[19]^

The process of bringing a new pharmaceutical drug to market typically spans a decade or longer. Throughout this period, the investment of resources and labor necessary to conduct cellular, animal, and clinical trials is substantial and often incalculable, with the outcomes of such investments remaining uncertain. Consequently, repurposing existing drugs to identify new therapeutic applications presents a viable and effective alternative strategy for addressing the challenges associated with new drug discovery. In recent years, there has been a notable increase in the utilization of Food and Drug Administration (FDA)-approved vaccines and drugs, indicating that discovering new applications for established drugs is an excellent approach to mitigating the aforementioned challenges.^[20]^ For instance, sildenafil, a drug traditionally used to treat erectile dysfunction in men, was discovered to have therapeutic potential for pulmonary hypertension and cardiovascular diseases (such as myocardial ischemia) in 2021.^[21]^

Utilizing advanced data mining techniques, a pivotal gene associated with both osteoporosis and sarcopenia was identified. Key genes were subsequently extracted and analyzed through the drug gene interaction database to pinpoint FDA-approved drugs capable of targeting these genes.

## 2. Materials and methods

### 2.1. Study design and data collection

The gene expression omnibus (GEO) serves as a public repository for functional genomics data, supporting data submissions that adhere to the Minimum Information About a Microarray Experiment standards. The platform provides tools to facilitate users in querying and downloading experimental data and curated gene expression profiles. Inclusion criteria for datasets necessitated the presence of independent arrays with substantial sample sizes and data pertinent to human subjects. Gene expression datasets were extracted from the GEO database utilizing the search terms “osteoporosis” and “sarcopenia.” As a result, 2 datasets, designated as GSE35958^[22]^ and GSE1428,^[23]^ were incorporated into the study. The GSE35958 dataset consisted of RNA-sequencing data from 5 patients, aged between 79 and 94 years, diagnosed with primary osteoporosis, alongside 4 healthy controls, aged between 79 and 89 years, all sourced from Wuerzburg. Concurrently, the GSE1428 dataset comprised RNA-sequencing data from vastus lateralis muscle samples collected from 12 individuals in Boston diagnosed with sarcopenia, aged between 70 and 80 years, as well as 10 young, healthy individuals aged between 19 and 25 years. Comparative analysis of these datasets revealed significant differences in gene expression levels between the 2 groups.

### 2.2. Identify differentially expressed genes (DEGs)

The datasets GSE35958 and GSE1428 underwent normalization, and the R packages “edgeR” and “limma” were utilized to identify DEGs between patient and control samples.^[24]^ For each gene, fold changes in expression were calculated. Genes exhibiting fold changes >1.2 and *P*-values <.05 were classified as DEGs. An overlap analysis was performed on the 2 DEG sets to identify genes common to both sarcopenia and osteoporosis. The R package “VennDiagram” was employed to determine these shared DEGs.^[25]^ Subsequently, the common genes were selected for further analysis.

### 2.3. Gene ontology (GO) enrichment analysis

The study employed the Database for Annotation, Visualization, and Integrated Discovery database (http://david.ncifcrf.gov/summary.jsp) to perform a GO enrichment analysis on the genes common to both osteoporosis and sarcopenia, encompassing the categories of biological process (BP), cellular component, and molecular function (MF).^[26]^ A more effective strategy involves granting access through queries to an integrated database that disseminates biologically comprehensive information across extensive datasets and offers graphical summaries of functional characteristics. The analysis results and graphical presentations exhibit dynamism through the integration of primary data and external data repositories, thereby providing comprehensive and extensive data coverage. In the GO enrichment analysis, the 8 genes with the lowest p-values were selected.

### 2.4. Reactome pathway analysis

The Reactome Knowledgebase (https://reactome.org), a core biological data resource within Elixir and the Global Core Biodata Resource, provides meticulously curated molecular details pertaining to a wide range of standard and disease-related BP. This resource is engaged in an ongoing review process aimed at the complete annotation of the human proteome.^[27]^ The study focuses on disease-causing genetic variants of proteins and small-molecule drugs within the context of biological pathways and supports the explicit annotation of cell- and tissue-specific pathways.

### 2.5. Protein–protein interaction (PPI) network and gene module analysis

The study utilizes the Search Tool for the Retrieval of Interacting Genes (STRING) database (http://string-db.org), an open-access resource designed to evaluate PPI data for shared genes. The STRING database, version 10.5, integrates text mining from PubMed and encompasses 9.6 million proteins from 2031 organism.^[28]^ Initially, we uploaded and mapped the gene list involved in GO enrichment analysis and Reactome pathways to the STRING platform. Subsequently, nodes that were not connected within the network were excluded based on the shared genes’ PPIs, with a minimum interaction score threshold set above 0.15, indicating low confidence. Thereafter, Cytoscape software was employed to construct PPI networks. Within Cytoscape, significant gene modules within the PPI networks were identified using the molecular complex detection (MCODE) algorithm. The parameters for MCODE were configured as follows: a degree cutoff exceeding 2, K-scores >2, and a node score cutoff above 0.2. Ultimately, the most significant gene modules from the PPI networks were selected for further validation analyses.

### 2.6. Quantitative real-time PCR (qRT-PCR) array validation

To corroborate the results of the bioinformatics analysis, muscle and bone tissues were collected from patients with osteoporosis and sarcopenia for qRT-PCR validation. The study included participants diagnosed with osteoporosis and sarcopenia (n = 12) and a healthy control group (n = 12), matched for age, sex, and body mass index. Participants in the osteoporosis and sarcopenia groups were recruited based on confirmed diagnoses through dual-energy X-ray absorptiometry scans for osteoporosis and the European Working Group on Sarcopenia in Older People criteria for sarcopenia. Exclusion criteria included individuals with secondary causes of bone loss (e.g., hyperparathyroidism, chronic steroid use) or muscle wasting (e.g., neuromuscular disorders, severe malnutrition). Ethical approval for the study was granted by the Biological and Medical Ethics Research Committee at Zhangzhou Health Vocational College (Approval number: 2024023), and written informed consent was obtained from each participant prior to enrollment.

Total RNA was extracted from tissue lesions using TRIzol reagent (Sangon Biotech Shanghai Co. Ltd, Shanghai, China). RNA samples were reverse transcribed into complementary DNA utilizing the First Strand cDNA Synthesis Kit (ABclonal, Wuhan, China), following the manufacturer’s protocol. Upon completion of the reverse transcription process, the reaction mixture was subjected to a 10-fold dilution. The thermal cycling conditions included an initial pre-denaturation step of 40 cycles of denaturation at 95 °C for 3 minutes, followed by 95 °C for 15 seconds and annealing/extension at 60 °C for 60 seconds. Primer sequences were validated using the NCBI BLAST tool and subsequently synthesized by Sangon Biotech Shanghai Co. Ltd. The relative expression levels of mRNA were quantified using the 2−ΔΔCT method, with the GAPDH gene serving as the internal reference. *P* < .05 was considered a statistically significant difference. The primer sequences are shown in Table 1.

**Table 1:** List of primer sequences used for quantitative real-time PCR.

Gene	Sequences	
	Forward	Reverse
AXL	GTGGGCAACCCAGGGAATATC	GTACTGTCCCGTGTCGGAAAG
GAB1	GATGGTTCGTGTTACGCAGTG	CGCTGTCTGCTACCAAGTAGAA
ERBB2	TGCAGGGAAACCTGGAACTC	ACAGGGGTGGTATTGTTCAGC
NRP2	CCAACGGGACCATCGAATCTC	CCAGCCAATCGTACTTGCAGT
ESR1	GAAAGGTGGGATACGAAAAGACC	GCTGTTCTTCTTAGAGCGTTTGA

### 2.7. Drug–gene interactions

The finalized list of genes served as potential targets in the search for existing pharmaceuticals or small organic compounds. The Drug–Gene Interaction Database (DGIdb; www.dgidb.org) is an online resource that amalgamates various data sources detailing drug–gene interactions and gene druggability.^[29]^ It offers a user-friendly graphical interface and a well-documented application programming interface for data querying. Similarly, the STITCH database (http://stitch.embl.de/) consolidates these diverse data sources for 430,000 chemicals into a unified, accessible resource.^[30]^ In conjunction with the expanded scope of the database, we have introduced a novel network visualization feature that permits users to examine the binding affinities of chemicals within the interaction network. This enhancement facilitates a rapid assessment of the potential impacts of a chemical on its interaction partners.

### 2.8. Statistical analysis

The statistical analyses conducted in this study utilized R software (version 4.4.1, https://www.r-project.org/). The normality of various parameters was evaluated using the Anderson-Darling test. Group differences were analyzed using the Student unpaired *t* test, with a *P*-value of <.05 deemed statistically significant.

## 3. Results

### 3.1. Identify DEGs

**Figure 1.**
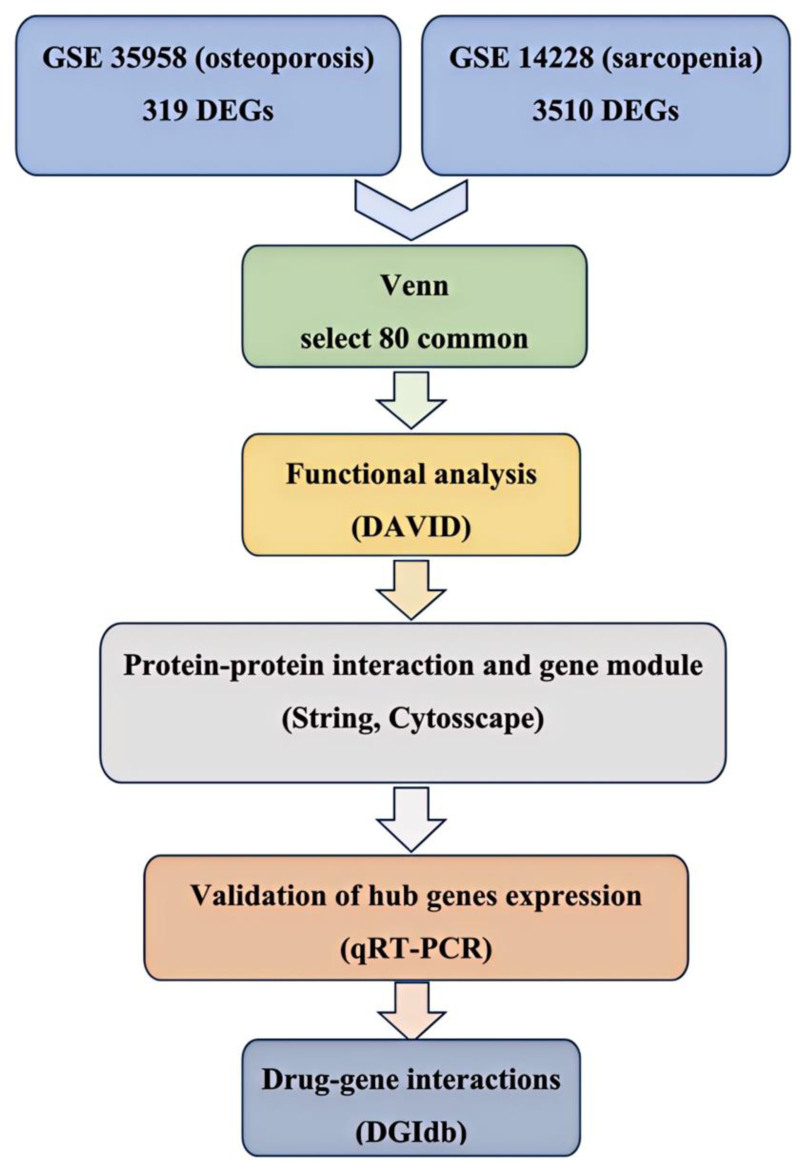
Overall process of strategy. Differentially expressed genes were obtained through GSE35958 (osteoporosis) and GSE1428 (sarcopenia), common differentially expressed genes were selected, and DAVID was used to analyze the function and gene ontology of the extracted genes. Further enrichment was performed using STRING and cytoscape molecular network analysis. Other databases were used to verify hub gene expression, and the drug–gene interaction database was used to determine the final enriched gene list and its interactions with known drugs. DAVID = Database for Annotation, Visualization, and Integrated Discovery, STRING = Search Tool for the Retrieval of Interacting Genes.

**Figure 2.**
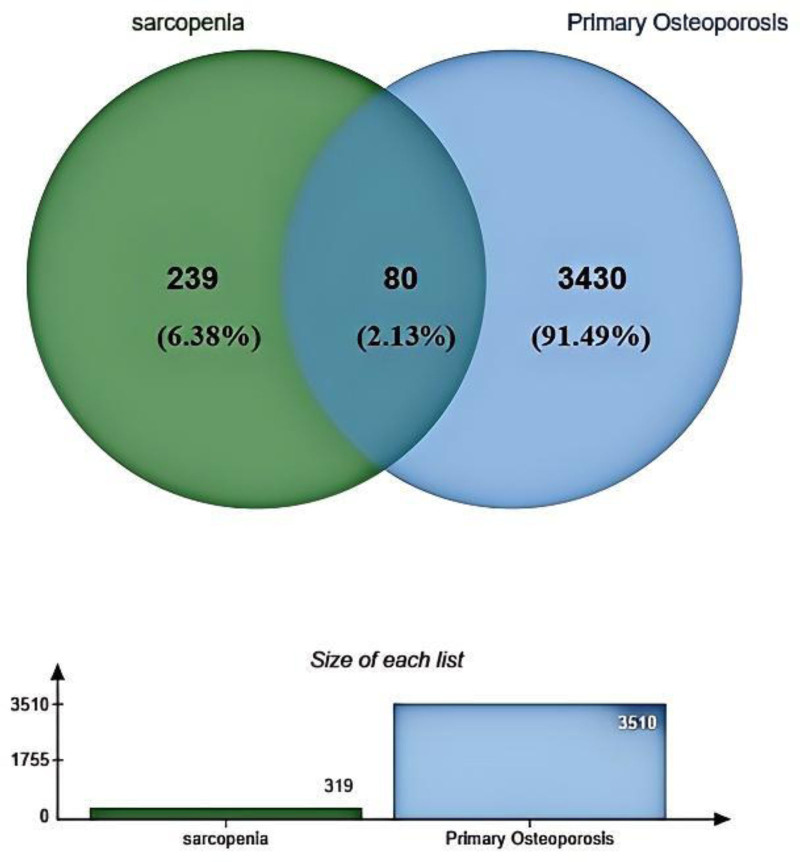
Venn diagram showing the superposed genes between primary osteoporosis and sarcopenia.

The research methodology employed in this project is delineated in the strategy outlined in Fig. 1. Data were sourced from 2 independent datasets, GSE35958 (pertaining to primary osteoporosis) and GSE1428 (pertaining to sarcopenia), both of which were retrieved from the GEO. The microarray data underwent normalization, followed by the identification of gene fragments. Subsequently, genes exhibiting a *P*-value of <.05 were selected, resulting in 319 genes from GSE35958 and 3510 genes from GSE1428. A Venn diagram analysis was conducted to identify 80 genes that were common to both datasets, as illustrated in Fig. 2.

### 3.2. GO enrichment analysis

**Figure 3.**
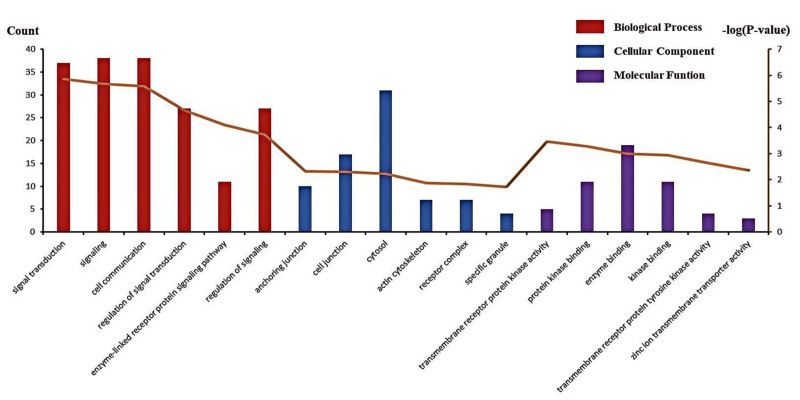
GO enrichment annotation analysis in the biological process (BP), cellular component (CC), and molecular function (MF) of those gene sets. GO = gene ontology.

The study reported the results of an analysis involving 80 common genes identified through GO enrichment annotation using the Database for Annotation, Visualization, and Integrated Discovery. Enriched BP annotations were selected, resulting in the identification of 6 groups of the most significant annotations: (1) signal transduction (*P* = 1.39 × 10^‐6^); (2) signaling (*P* = 2.11 × 10^‐6^); (3) cell communication (*P* = 2.61 × 10^‐6^); (4) regulation of signal transduction (*P* = 2.20 × 10^-5^); (5) enzyme-linked receptor protein signaling pathway (*P* = 8.03 × 10^‐5^); and (6) regulation of signaling (*P* = 1.91 × 10^-4^). These groups comprised 37, 38, 38, 27, 11, and 27 genes from the query set, respectively, as detailed in Table 2. Osteoporosis and sarcopenia represent intricate BP associated with human aging. Intervening in these critical life processes may mitigate the progression of related diseases to some degree. Following the onset of osteoporosis and sarcopenia, drug therapy can promptly and concurrently activate multiple biological pathways. Consequently, the relatively low *P*-values underscore the relevance of these BP annotations. Cellular component analysis revealed that the majority of genes were expressed in the anchoring junction, cell junction, cytosol, and actin cytoskeleton. Additional details are provided in Table 3. Through the analysis of molecular functional annotation, 6 pathways were identified. Of these, the most significant MF is transmembrane receptor protein kinase activity (*P* = 3.41 × 10^‐04^), encompassing 5 genes, as detailed in Table 4. Graphical representations of the GO enrichment analysis, including cellular components, BP, and MF, were generated using R statistical software (version 4.4.1), as illustrated in Fig. 3.

**Table 2:** Summary of biological process genes set GO enrichment analysis.

Term	Count	*P*-value	Genes
Signal transduction	37	1.39E‐06	DOCK5, KHDRBS1, NRP2, BMPR2, DAGLA, CUL5, PRKCSH, INSIG1, ZFP36L1, OR2S2, CASP10, SLC39A9, ERBB2, PPFIA1, SPX, CHML, EPHB2, SKIL, RALGPS2, SPAG9, SPEN, PPP1R12A, MCF2, CLEC11A, PLCL2, GAB1, PAWR, MYO9A, PRLR, ESR1, PML, APBB1IP, TRAF4, AXL, TXNIP, RAPGEF5, CDK14
Signaling	38	2.11E‐06	DOCK5, KHDRBS1, NRP2, BMPR2, DAGLA, CUL5, PRKCSH, INSIG1, SLC1A4, ZFP36L1, OR2S2, CASP10, SLC39A9, ERBB2, PPFIA1, SPX, CHML, EPHB2, SKIL, RALGPS2, SPAG9, SPEN, PPP1R12A, MCF2, CLEC11A, PLCL2, GAB1, PAWR, MYO9A, PRLR, ESR1, PML, APBB1IP, TRAF4, AXL, TXNIP, RAPGEF5, CDK14
Cell communication	38	2.61E‐06	DOCK5, KHDRBS1, NRP2, BMPR2, DAGLA, CUL5, PRKCSH, INSIG1, SLC1A4, ZFP36L1, OR2S2, CASP10, SLC39A9, ERBB2, PPFIA1, SPX, CHML, EPHB2, SKIL, RALGPS2, SPAG9, SPEN, PPP1R12A, MCF2, CLEC11A, PLCL2, GAB1, PAWR, MYO9A, PRLR, ESR1, PML, APBB1IP, TRAF4, AXL, TXNIP, RAPGEF5, CDK14
Regulation of signal transduction	27	2.20E‐05	BMPR2, CANT1, INSIG1, CASP10, PEG10, ERBB2, SLC39A8, EPHB2, SKIL, SPAG9, MCF2, PLCL2, PAWR, MYO9A, PRLR, ESR1, PML, PPM1A, ELF1, SIKE1, MDFIC, TRAF4, AXL, CLDN18, TRIM15, CDK14, LLGL1
Enzyme-linked receptor protein signaling pathway	11	8.03E‐05	NRP2, BMPR2, CUL5, AXL, ERBB2, GAB1, TXNIP, EPHB2, SKIL, PRLR, PML
Regulation of signaling	27	1.91E‐04	BMPR2, CANT1, INSIG1, CASP10, PEG10, ERBB2, SLC39A8, EPHB2, SKIL, SPAG9, MCF2, PLCL2, PAWR, MYO9A, PRLR, ESR1, PML, PPM1A, ELF1, SIKE1, MDFIC, TRAF4, AXL, CLDN18, TRIM15, CDK14, LLGL1

GO = gene ontology.

**Table 3:** Summary of cell component genes set GO enrichment analysis.

Term	Count	*P*-value	Genes
Anchoring junction	10	4.83E-03	APBB1IP, DOCK5, BMPR2, PPP1R12A, TRAF4, PPFIA1, GAB1, CLDN18, TRIM15, LLGL1
Cell junction	17	0.004949879	DOCK5, NRP2, DAGLA, BMPR2, PPP1R12A, GAB1, SLC1A4, MYO9A, APBB1IP, TRAF4, ERBB2, PPFIA1, CLDN18, TRIM15, EPHB2, SPTAN1, LLGL1
Cytosol	31	0.005799508	SETD4, DOCK5, KHDRBS1, DTYMK, SERPINB13, CUL5, ANKRD12, ZFP36L1, CASP10, PEG10, ERBB2, PPFIA1, CHML, EPHB2, SPTAN1, CEP290, SPAG9, PPP1R12A, SMOX, MCF2, GAB1, PAWR, MYO9A, ESR1, PML, APBB1IP, PPM1A, SIKE1, TRAF4, TXNIP, CDK14
Actin cytoskeleton	7	0.013108988	DOCK5, PPP1R12A, AXL, PAWR, SPTAN1, MYO9A, LLGL1
Receptor complex	7	0.014768738	APBB1IP, NRP2, BMPR2, TRAF4, AXL, ERBB2, PRLR
Specific granule	4	0.018630177	CANT1, ATP11A, SPTAN1, CEP290

GO = gene ontology.

**Table 4:** Summary of molecular function genes set GO enrichment analysis.

Term	Count	*P*-value	Genes
Transmembrane receptor protein kinase activity	5	3.41E‐04	NRP2, BMPR2, AXL, ERBB2, EPHB2
Protein kinase binding	11	5.18E‐04	SPAG9, KHDRBS1, SIKE1, BMPR2, PPP1R12A, TRAF4, PRKCSH, ERBB2, PRLR, ESR1, LLGL1
Enzyme binding	19	9.85E‐04	SPAG9, KHDRBS1, DOCK5, BMPR2, PPP1R12A, SERPINB13, CUL5, PRKCSH, PAWR, PRLR, ESR1, PML, SIKE1, TRAF4, CASP10, ERBB2, TXNIP, CHML, LLGL1
Kinase binding	11	0.001161511	SPAG9, KHDRBS1, SIKE1, BMPR2, PPP1R12A, TRAF4, PRKCSH, ERBB2, PRLR, ESR1, LLGL1
Transmembrane receptor protein tyrosine kinase activity	4	0.00225914	NRP2, AXL, ERBB2, EPHB2
Zinc ion transmembrane transporter activity	3	0.00431059	SLC39A9, SLC11A2, SLC39A8

GO = gene ontology.

### 3.3. Reactome pathway analysis

**Figure 4.**
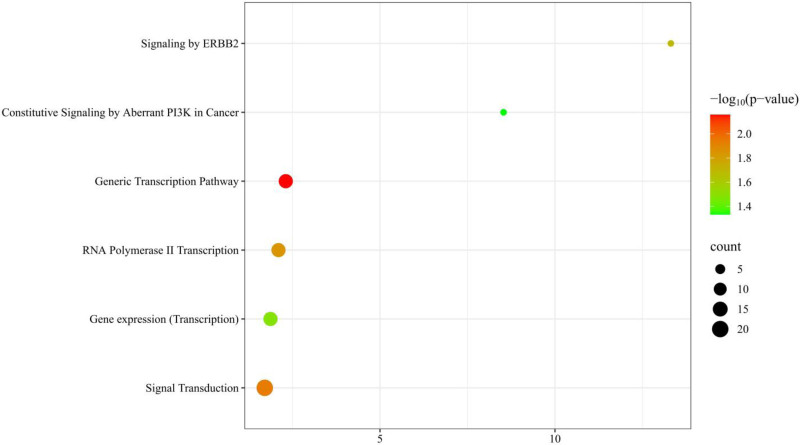
Enriched biological Reactome pathway analysis.

The study identified 6 pathways with a *P*-value <.05 using the Reactome pathway enrichment analysis program, indicating a significant association with osteoporosis and sarcopenia (Table 5). The 6 most enriched biological Reactome pathways were: (1) Generic Transcription Pathway (*P* = 6.97 × 10^‐3^), (2) Signal Transduction (*P* = .011), (3) RNA Polymerase II Transcription (*P* = .014), (4) Signaling by ERBB2 (*P* = .020), (5) Gene Expression (Transcription) (*P* = .033), and (6) Constitutive Signaling by Aberrant PI3K in Cancer (*P* = .0464). The pathways comprised 13, 20, 13, 3, 13, and 3 genes pertinent to the pathway enrichment analysis, respectively. To illustrate these details comprehensively, we employed R statistical software (version 4.4.1) for mapping purposes (see Fig. 4). In total, 65 associated genes were identified through GO enrichment analysis and 6 through Reactome pathway analysis. These genes will subsequently be utilized in the PPI analysis.

**Table 5:** Summary of Reactome pathway enrichment analysis.

Term	Count	*P*-value	Genes
Generic transcription pathway	13	0.006966446	SERPINB13, TOP3A, COX5B, ESR1, PML, PPM1A, ELF1, ZKSCAN7, CASP10, ERBB2, TXNIP, SKIL, ZNF124
Signal transduction	20	0.01161327	SPEN, KHDRBS1, DOCK5, NRP2, DAGLA, BMPR2, PPP1R12A, CUL5, MCF2, GAB1, MYO9A, ESR1, PML, APBB1IP, PPM1A, CASP10, AXL, ERBB2, SKIL, SPTAN1
RNA polymerase II transcription	13	0.01441148	SERPINB13, TOP3A, COX5B, ESR1, PML, PPM1A, ELF1, ZKSCAN7, CASP10, ERBB2, TXNIP, SKIL, ZNF124
Signaling by ERBB2	3	0.020468634	CUL5, ERBB2, GAB1
Gene expression (transcription)	13	0.032938261	SERPINB13, TOP3A, COX5B, ESR1, PML, PPM1A, ELF1, ZKSCAN7, CASP10, ERBB2, TXNIP, SKIL, ZNF124
Constitutive signaling by aberrant PI3K in cancer	3	0.046422275	ERBB2, GAB1, ESR1

### 3.4. PPI network and gene module analysis

**Figure 5.**
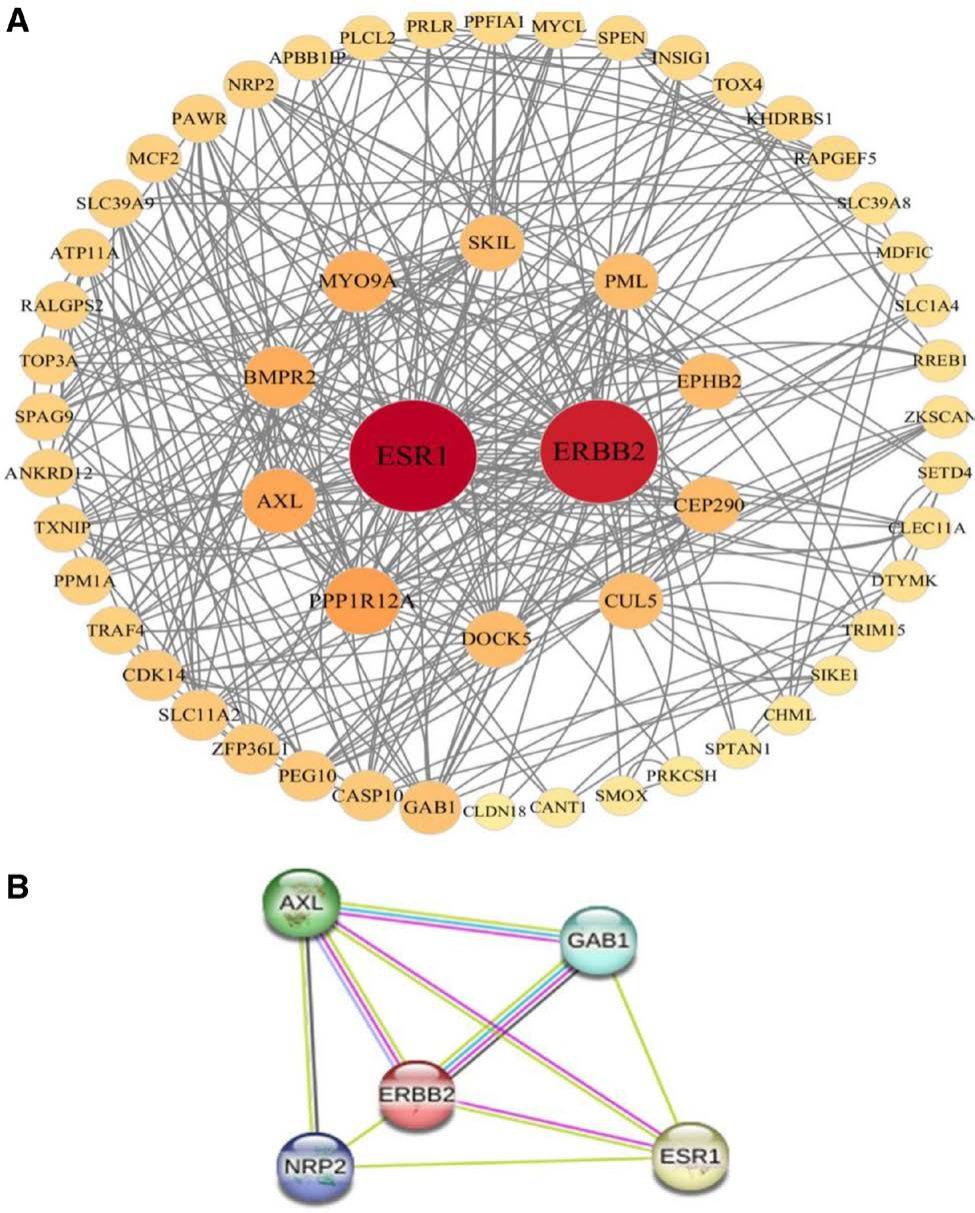
Protein–protein interaction (PPI) network. (A) Protein–protein interaction (PPI) network of the superposed genes between osteoporosis and sarcopenia. (B) Tightest module from the PPI network.

PPI analysis was conducted utilizing the STRING database. In this study, the parameters were configured to a minimum required interaction score of low confidence (0.15), with hidden nodes included in the network. The network statistics revealed 65 nodes and 162 edges, with an average node degree of 4.98, a local clustering coefficient of 0.289, and a PPI enrichment *P*-value of 1.872 × 10^‐6^ (Fig. 5A). The observed network exhibits a significantly greater number of interactions than anticipated. This suggests that the proteins engage in more frequent interactions among themselves compared to what would be expected from a random selection of proteins of equivalent size and degree distribution derived from the genome. This enrichment indicates that the proteins, as a collective, possess at least partial biological connectivity. Subsequently, the Cytoscape software was utilized to construct the PPI network. The MCODE algorithm was applied to identify essential gene modules within the PPI network, with parameters set as follows: degree cutoff > 2, K-scores > 2, and node score cutoff > 0.2.

Finally, the study selected 5 central genes (AXL, GAB1, ERBB2, NRP2, ESR1) that formed the most tightly connected network of modules according to the above criteria (Fig. 5B).

### 3.5. The hub genes’ qRT-PCR validation

**Figure 6.**
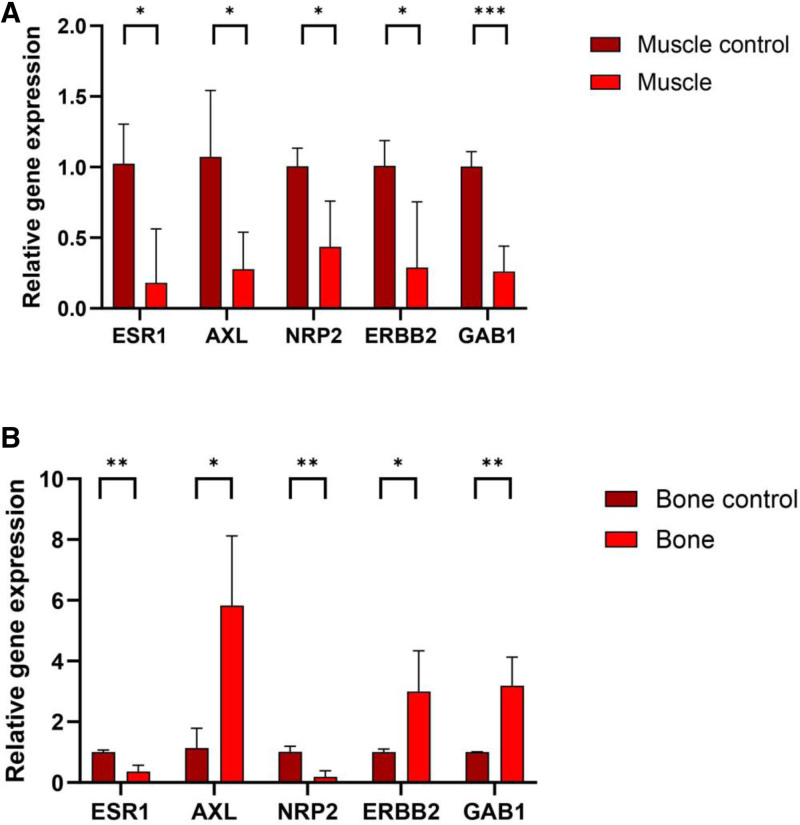
The hub genes validation by qRT-PCR. (A) Genes expression in muscle. (B) Genes expression in bone. qRT-PCR = quantitative real-time PCR.

A qRT-PCR methodology was employed to assess the expression levels of 5 candidate genes. The validation results demonstrated a significant reduction in the muscle expression levels of AXL, GAB1, ERBB2, NRP2, and ESR1 in the samples (*P* < .05, Fig. 6A), corroborating the reliability of the bioinformatics-derived analytical signaling pathway findings. Additionally, the validation results revealed a significant increase in the bone expression levels of AXL, ERBB2, and GAB1, whereas ESR1 and NRP2 exhibited a significant decrease (Fig. 6B). These findings suggest that the selected genes exert a measurable impact within the studied context on both sarcopenia and osteoporosis.

### 3.6. Drug–gene interactions

**Figure 7.**
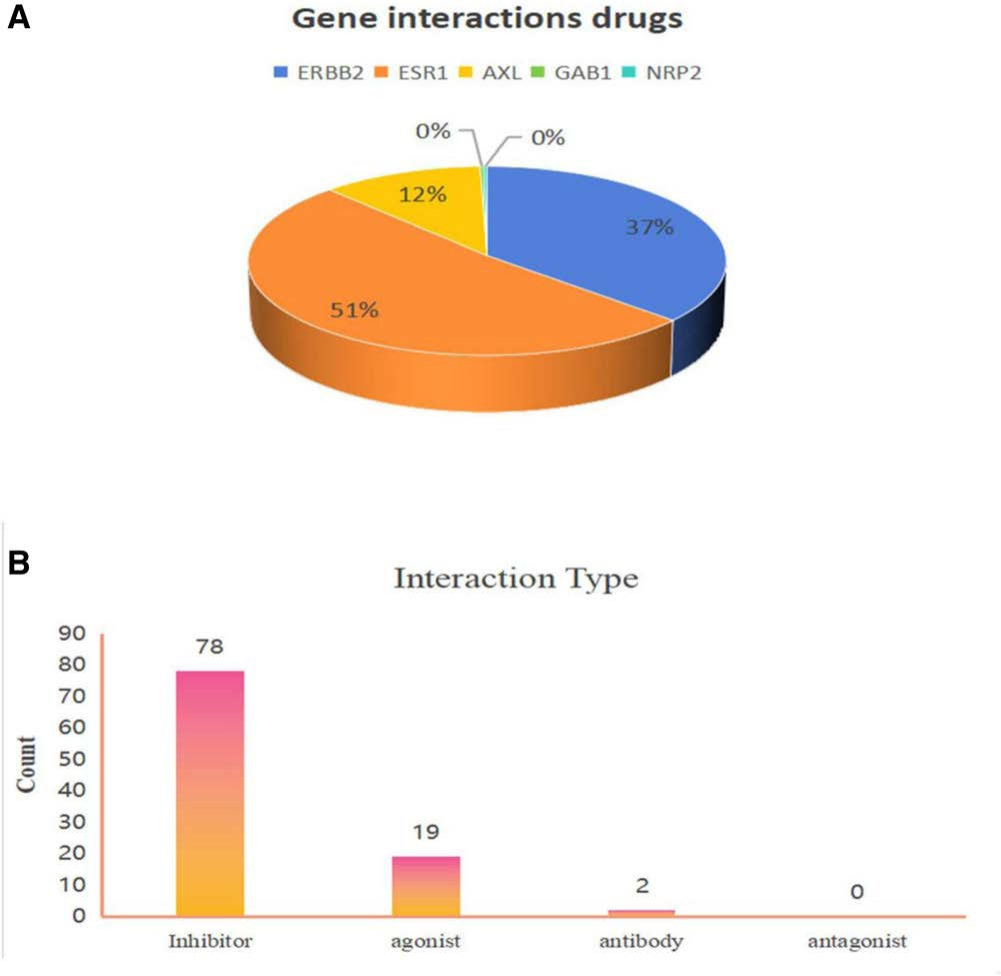
Drug type. (A) Genes interactions the proportion of drugs taken. (B) Drugs interaction type count.

**Figure 8.**
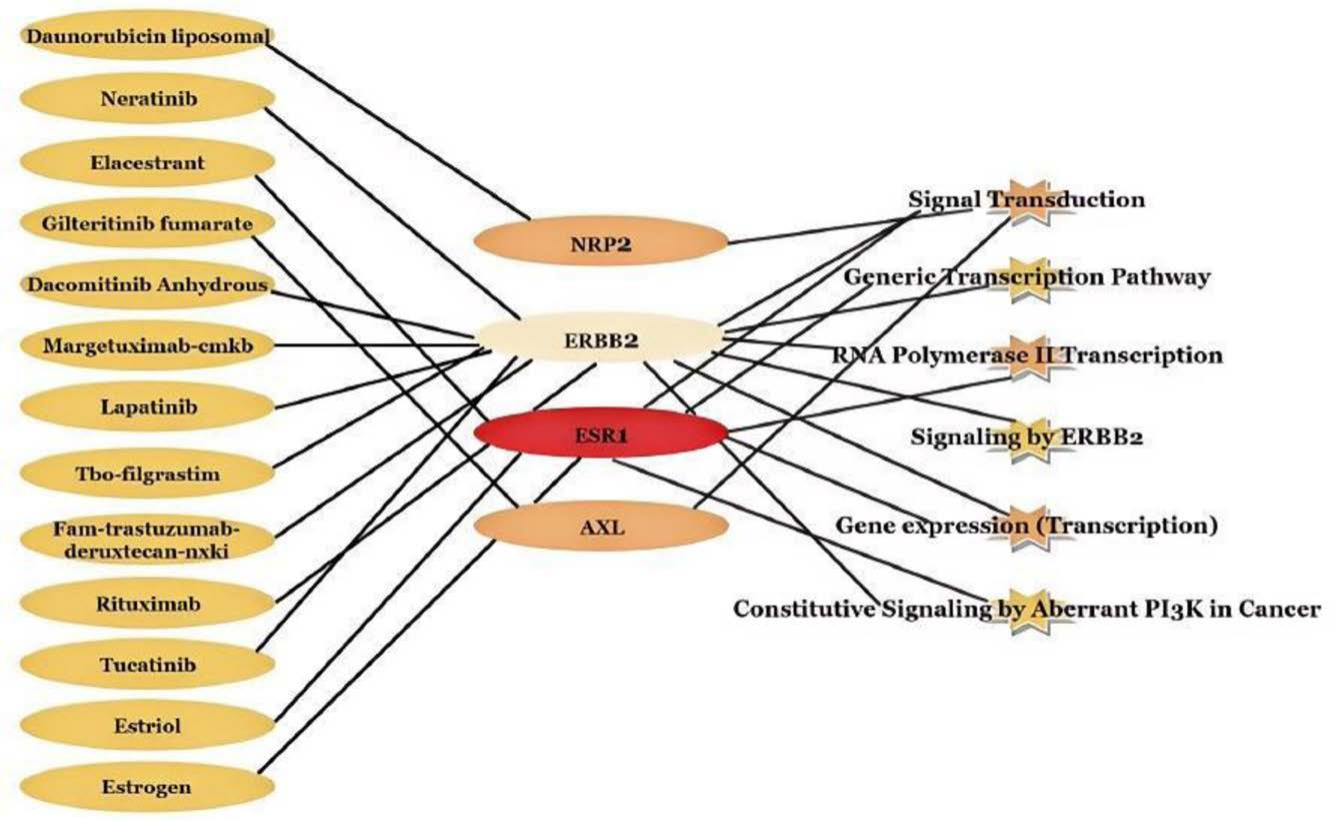
Interrelation of 13 drugs with genes and pathways.

Finally, we conducted a drug-gene interaction analysis for the 5 genes identified by the module, revealing that 416 drugs exert specific effects on these genes and associated pathways. The potential gene targets identified include ERBB2 (targeted by 152 drugs), ESR1 (214 drugs), AXL (48 drugs), and both GAB1 and NRP2 (2 drugs each) (Fig. 7A). Our study identified a total of 78 inhibitors, 19 agonists, and 2 antibodies (Fig. 7B). A drug with associated PubMed IDs was selected, and its drug-gene interactions were evaluated, demonstrating that it has been studied to a certain extent and has received FDA approval. Table 6 enumerates 13 pharmacological agents proposed as potential therapeutic interventions for osteoporosis and sarcopenia. These include 7 antineoplastic agents, 2 drugs for postmenopausal symptoms, one agent for the treatment of neutropenia, one hormone replacement agent, and one binder. The gene-pathway associations pertinent to these 13 drugs are depicted in Fig. 8.

**Table 6:** Selected drugs for osteoporosis and sarcopenia (approved by FDA).

Drug	Description	Gene	Drug class	Interaction score	Reference (PubMed ID)
Daunorubicin liposomal	Antineoplastic agent	NRP2	Small molecule/chemotherapy	1.154	18451141, 30504411, 18019828, 31213803, 30511323
Neratinib	Antineoplastic agent	ERBB2	Small molecule/kinase inhibitors/ERBB2 inhibitor	0.62	34077739, 27597738, 29967253, 30500458, 37269887, 34126793
Elacestrant	Treatment of postmenopausal symptoms	ESR1	Small molecule	0.61	35584336, 37060385, 37871699, 39087959, 33513026, 38381994
Gilteritinib fumarate	Inhibitor	AXL	NA	0.55	39268560, 35496349, 33390479
Dacomitinib Anhydrous	Antineoplastic agent	ERBB2	Small molecule/kinase inhibitors/pan ERBB inhibitor/pan ERBB inhibitor	0.46	NA
Margetuximab-cmkb	Antineoplastic agent	ERBB2	Monoclonal antibody	0.41	33821931, 33355736, 33761116, 37170847, 33459118, 34077236, 34916216
Lapatinib	Antineoplastic agent	ERBB2	Small molecule/ERBB2 mAb inhibitor/protein kinase inhibitors/chemotherapy	0.37	3660278, 20542996, 37883083, 35027594, 17229773, 33122343
Tbo-filgrastim	Treatment of neutropenia	ERBB2	Antineutropenic agents/recombinant protein	0.35	24352574, 21826527
Fam-trastuzumab-deruxtecan-nxki	Binder	ERBB2	NA	0.35	33753456, 36780610, 33629601, 37577308, 38970465, 35909271, 32282173, 34424404
Rituximab	Antineoplastic agent	ERBB2	Monoclonal antibody/ERBB2 mAb inhibitor/therapeutic antibodies	0.29	32403958, 25911943, 35542978, 21300696
Tucatinib	Antineoplastic agent	ERBB2	Small molecule/kinase inhibitors	0.26	36454580, 36454580, 36454580, 36454580, 34954044, 37227737, 37227708, 32548668
Estriol	Hormone replacement agents	ESR1	Small molecule/hormone replacement agents	0.25	23313336, 24529907, 35676404, 32788222, 28743541
Estrogen	Treatment of female sexual dysfunction, contraceptive, symptomatic treatment of menopausal symptoms	ESR1	Small molecule	0.25	33430527, 23994581, 25682174, 37047814, 37500767, 38625747, 37193881, 38330990, 32169631

## 4. Discussion

Emerging evidence indicates that a substantial proportion of individuals with osteoporosis experience fatigue, nutritional deficiencies, and frequent pathologic fractures, contributing to diminished ambulatory capacity and muscle strength. Both sarcopenia and osteoporosis are linked to the aging process, with sarcopenia being more prevalent among osteoporosis patients. These individuals often exhibit accelerated muscle atrophy, exacerbating impairments in various physiological functions. There may be common or overlapping pathogenic mechanisms underlying osteoporosis and sarcopenia. It is essential to elucidate these mechanisms to devise effective therapeutic strategies. Emerging evidence suggests that these conditions share common pathogenic mechanisms, including dysregulation of gene expression, impaired signal transduction, and altered cellular metabolism. This study identified key genes (ESR1, NRP2, AXL, ERBB2, and GAB1) and FDA-approved drugs (e.g., elacestrant, daunorubicin liposomal, and estriol) as potential therapeutic candidates for these conditions, providing new insights into their molecular underpinnings and treatment strategies.

GO and Reactome enrichment analyses revealed significant enrichment of these genes in pathways related to generic transcription, signal transduction, and RNA polymerase II transcription. The generic transcription pathway plays a critical role in regulating genes associated with aging, muscle weakness, and bone loss.^[31]^ Enhanced VEGF signal transduction has been shown to mitigate age-associated capillary degeneration, improve organ perfusion, and alleviate conditions such as hepatic steatosis, sarcopenia, and osteoporosis.^[32]^ The regulation of gene expression, which is fundamental to the development and survival of all organisms, commences with the transcription process catalyzed by RNA polymerase. RNA polymerase II transcription involves the unwinding of promoter DNA, initiation of de novo messenger RNA synthesis, transition to productive elongation, and ultimately terminates transcription, which is implicated in synthesizing most of the substances involved in osteoporosis and sarcopenia.^[33]^

In the findings of this study, qRT-PCR validation conducted on patients with sarcopenia and osteoporosis revealed a reduction in the expression of the ESR1 and NRP2 genes in both bone and muscle tissues. Conversely, the expression of AXL, ERBB2, and GAB1 was observed to decrease in muscle tissue while increasing in bone tissue. In the context of ESR1-targeted therapeutics, Elacestrant was identified as having the most favorable interaction score. Elacestrant (RAD-1901), a selective estrogen receptor (ER) degrader, received approval from the U.S. Food and Drug Administration on January 27, 2023, for the treatment of ER-positive, HER2-negative advanced breast cancer.^[34]^ Recent studies have also demonstrated its positive effects in breast cancer patients harboring ESR1 mutations.^[35]^ Elacestrant, as the inaugural oral selective ER degrader, demonstrates superior advantages and specificity compared to existing anti-osteoporosis medications, particularly in the treatment of osteoporosis in females. This is notably significant for patients with ESR1 mutations, who exhibit a relatively high prevalence and therapeutic response. Elacestrant functions as a dose-dependent mixed ER agonist/antagonist, exhibiting direct ER antagonistic properties and selectively down-regulating ER at elevated doses. It undergoes primary metabolism via the cytochrome P450 3A4 enzyme in the liver and is predominantly excreted through fecal elimination. In consideration of its clearance pathways, a reduction in dosage is advised for patients with moderate hepatic insufficiency; however, such adjustments are not necessary for patients with renal insufficiency.^[36]^

Additionally, daunorubicin liposomal, an antitumor agent characterized by reduced expression in bone and muscle tissues and possessing the highest interaction score, has shown efficacy in achieving targeted therapeutic effects through liposomal encapsulation. Liposome encapsulation facilitates enhanced cellular drug delivery and, along with complex hydrogels, has been demonstrated to mediate the reprogramming of osteosarcoma fibroblasts.^[37]^ Consequently, it holds potential as a therapeutic agent for sarcopenia. Concurrently, research has substantiated that the development of nano-loaded materials can induce apoptosis and augment immune responses through the inhibition of autophagy and the repolarization of macrophages.^[38]^ Given that osteoblast apoptosis in osteoporosis is partially attributed to increased osteoclast activity, these nano-loaded materials also represent a promising therapeutic avenue for osteoporosis treatment. The screening of hormone analogs, including Estriol, has been suggested for potential application in addressing postmenopausal osteoporosis and sarcopenia. The metabolic impact of estriol on bone and skeletal muscle elucidates cytokine-mediated bone-muscle crosstalk, which may be modulated in the absence of estrogen.^[39]^

The findings of this study underscore the potential of drug repurposing as a cost-effective and efficient strategy for addressing osteoporosis and sarcopenia. Elacestrant, daunorubicin liposomal, and estriol emerged as promising candidates for further investigation, with the potential to improve clinical outcomes for patients with these conditions. The identification of ESR1, NRP2, AXL, ERBB2, and GAB1 as key genes provides novel therapeutic targets and highlights the importance of understanding shared molecular pathways in osteoporosis and sarcopenia. However, this study has certain limitations. Its retrospective design, reliance on a gene-expression dataset with a relatively small sample size, and potential oversight of critical genes during the selection process may have introduced bias. To address these limitations, future research should focus on elucidating the molecular mechanisms of the identified drugs using cellular and animal models to confirm their efficacy and safety. Additionally, clinical trials are warranted to evaluate the therapeutic potential of Elacestrant, daunorubicin liposomal, and estriol in patients with osteoporosis and sarcopenia. Further exploration of nano-loaded materials for targeted drug delivery and the role of cytokine-mediated bone-muscle crosstalk in disease pathogenesis could enhance therapeutic outcomes. By pursuing these directions, future studies can build on the current findings to develop innovative and effective therapies, ultimately improving the quality of life for individuals affected by these conditions.

## 5. Conclusion

In conclusion, our study identified common DEGs associated with osteoporosis and sarcopenia, and we conducted a comprehensive analysis of their functions and interactions through enrichment analysis and PPI networks. The findings indicate that these 2 diseases share numerous pathogenic pathways potentially regulated by the identified hub genes, thereby elucidating possible mechanisms underlying both conditions. Furthermore, our research highlights novel candidate genes that may serve as biomarkers or potential therapeutic targets. The correlation study, along with the analysis of potential drugs targeting these genes, offers a novel direction for drug repurposing and the exploration of therapeutic pathways.

## Acknowledgments

We thank all the participants who supported our study. In particular, thanks to the GEO database and R software for the analytical data.

## Author contributions

**Conceptualization:** Zhangxin Chen, Jianxing Lin, Zhengjin Wang.

**Data curation:** Zhangxin Chen, Jianxing Lin, Zhengjin Wang.

**Formal analysis:** Zhangxin Chen, Jianxing Lin.

**Funding acquisition:** Zhangxin Chen, Jianxing Lin.

**Investigation:** Zhangxin Chen, Jianxing Lin.

**Methodology:** Zhangxin Chen, Jianxing Lin.

**Project administration:** Zhangxin Chen, Jianxing Lin.

**Resources:** Zhangxin Chen, Zhengjin Wang.

**Software:** Zhangxin Chen, Zhengjin Wang.

**Supervision:** Zhangxin Chen, Zhengjin Wang.

**Validation:** Zhangxin Chen, Zhengjin Wang.

**Visualization:** Zhangxin Chen, Zhengjin Wang.

**Writing – original draft:** Zhangxin Chen, Zhengjin Wang.

**Writing – review & editing:** Zhangxin Chen, Zhengjin Wang.
